# Archived Cytogenetic Cell Pellets Used to Detect a BCR::ABL1 Driver Mutation Eight Years before Disease Presentation

**DOI:** 10.1155/2024/2127657

**Published:** 2024-03-20

**Authors:** Ramakrishnan Sasi, Michelle Spruill, Peter L. Perrotta

**Affiliations:** Department of Pathology, Anatomy and Laboratory Medicine, West Virginia University, Morgantown, WV 26506, USA

## Abstract

Evidence suggests that the earliest genetic events in the evolution of a cancer can predate diagnosis by several years or decades. In chronic myeloid leukemia (CML), the BCR::ABL1 fusion driver mutation can be present for an extended period before clinical disease manifests. The time between the BCR::ABL1 occurrence and symptom onset is referred to as the latency period. Though modeling studies predict this latency period is no more than ten years, it is still unclear how long it can be. We present a case of a patient referred for suspected CML. Both karyotype and FISH analysis identified the *t*(9;22)(*q*34;*q*11.2) translocation resulting in the Philadelphia chromosome formation in 98.5% of cells analyzed. The patient responded to imatinib and achieved a sustained complete hematologic and cytogenetic remission. Clinical history revealed that the same patient presented eight years previously with anemia. Various non-neoplastic conditions were excluded, and a bone marrow biopsy was performed to rule out MDS. Cytogenetic analysis at that time revealed del(20*q*) as the sole abnormality in all 20 cells analyzed. No treatment was given since the presence of isolated del(20*q*) is not considered evidence of MDS in the absence of diagnostic morphologic criteria. Retrospective FISH analysis of archived bone marrow pellets from this previous specimen revealed the presence of BCR::ABL1 in 1.8% of cells. A clonal population of cells harboring the BCR::ABL1 fusion was unambiguously detected in this patient's archived bone marrow pellet obtained eight years before the current CML diagnosis. This case demonstrates that Carnoy's fixed nuclear pellets stored in cytogenetic laboratories are suitable for detecting driver mutations years before disease presentation. Such archived material may be useful for the retrospective studies needed to better understand the initiation and subsequent development of hematological malignancies. By identifying individuals who are at increased risk, it may be possible to initiate preventive measures or begin treatment at an earlier stage before disease progression.

## 1. Introduction

We present a case in which low-level BCR::ABL1 fusion was discovered in a patient years before presenting with chronic myelogenous leukemia (CML). At the time of diagnosis, 90–95% of patients with CML have cytogenetic evidence of the Philadelphia chromosome [(der)(22)*t*(9;22)(*q*34.1;*q*11.2)] [1]. This unique genetic aberration arises as a consequence of a reciprocal translocation situating the ABL1 gene from chromosome 9 next to the BCR gene on chromosome 22. This BCR::ABL1 gene fusion product is the defining molecular lesion of CML and is also found in a subset of acute lymphoblastic leukemia [1, 2].

Generally, at the time of initial CML diagnosis, BCR::ABL1 fusion is found in >90% of interphase nuclei by fluorescence in situ hybridization (FISH) [3], and the *t*(9;22)(*q*34;*q*11.2) translocation is seen in virtually 100% of G-banded metaphase cells unless the fusion is due to a variant rearrangement such as a cryptic insertion or a three- or four-way translocation. What causes a hematopoietic clone carrying the BCR::ABL1 fusion to expand to this high level after years of latency is not understood.

Studies of survivors of the atomic bombings of Hiroshima and Nagasaki who later developed CML suggest a mean latency of eight years between BCR::ABL1 induction and clinical presentation [4, 5]. However, the presence of driver mutations in normal tissues, including blood from healthy individuals with clonal hematopoiesis, some of whom subsequently develop malignancies, supports a more extended multi-hit pathway beginning very early in life [6].

It is now accepted that several mutations are required for cancer formation [7, 8]. Mutated cell proliferation is commonly seen in association with aging and/or in response to environmental insults and chronic inflammation could affect the rate of clonal expansion [9]. Mutations in cancer-associated genes drive tumor outgrowth, but our knowledge of the timing of driver mutations and subsequent clonal dynamics is limited [10, 11]. Recent publications have generated excitement showing possible timelines for clonal evolution that include the accumulation of driver mutations over the lifespan, some even occurring in utero [6, 8, 9, 11, 12].

To the best of our knowledge, there has not been a retroactive case study using FISH on archived bone marrow cytogenetic cell pellets demonstrating that the key BCR::ABL1 driver mutation existed for many years in a patient before the development of CML.

## 2. Case Presentation

A 57-year-old female patient was referred to the hematology clinic with anemia, fatigue, malaise, weight loss, night sweating, and abdominal fullness. The patient had no known family history of cancer, and there was no evidence of environmental exposure to known carcinogens. The white blood cell count was above 90,000/*µ*L and the platelet count above 550,000/*μ*L. The CBC showed absolute neutrophilia and normocytic anemia. Peripheral blood flow cytometry showed left-shifted neutrophils and no blasts. Bone marrow biopsy was hypercellular for patient age due to myeloid hyperplasia. Rare dyspoiesis was identified in the erythroid precursors. Several small and hyperlobulated megakaryocytes were present. No increase in blasts was identified. A diagnosis of CML was made based on the presence of the der(22)*t*(9;22)(*q*34;*q*11.2) in all twenty metaphases analyzed and BCR::ABL1 fusion found in 99.5% of interphases by FISH.

Review of the electronic medical record revealed the patient was seen eight years prior for anemia and mild cytopenia. Anemia and cytopenia may be associated with a variety of non-neoplastic conditions such as iron deficiency, vitamin B-12 and folate deficiencies, alcoholism, liver diseases, hypothyroidism, gastric diseases and gastrectomy, parvovirus infection, chemotherapy, and drug/toxin-induced disorders of DNA synthesis and replication. These were all etiologically excluded. A bone marrow biopsy was performed to exclude a myelodysplastic syndrome (MDS). This bone marrow was normocellular with no evidence of a myeloid neoplasm, acute leukemia, or marrow infiltrative process. Differential counts on 300 cells showed no increase in granulocytes. Both blasts and basophils were within the normal range (less than 1%).

Karyotype analysis and FISH studies at that time revealed a 20*q* deletion as the sole abnormality present in all twenty cells analyzed. The patient's condition resolved, and no treatment was provided since the presence of isolated del(20*q*) is not considered evidence of MDS in the absence of diagnostic morphologic criteria as defined by the World Health Organization (WHO) [2], though a low-grade or evolving MDS could not be entirely excluded.

## 3. Results

### 3.1. Clinical Cytogenetic Testing

Bone marrow aspirates were cultured unstimulated overnight and for 48 h according to routine cytogenetic protocols. The cultured cell pellets were prepared and preserved in Carnoy's fixative (3:1 methanol : acetic acid) and stored at −80°C.

Chromosome analysis was performed on twenty G-banded metaphase cells, and the resulting karyotypes were described according to the International System for Human Cytogenetic Nomenclature (ISCN 2020) [13]. FISH was performed according to standard procedures; briefly, nuclear cell pellets were washed twice in Carnoy's fixative, dropped onto glass microscope slides which were incubated in 2XSSC, and dehydrated with a 75%, 85%, and 95% alcohol series. Hybridization was carried out using the Vysis LSI BCR/ABL1 Tri-Color, Dual Fusion Translocation Probe (Abbott Molecular) according to the manufacturer's instructions.

**Figure 1:**
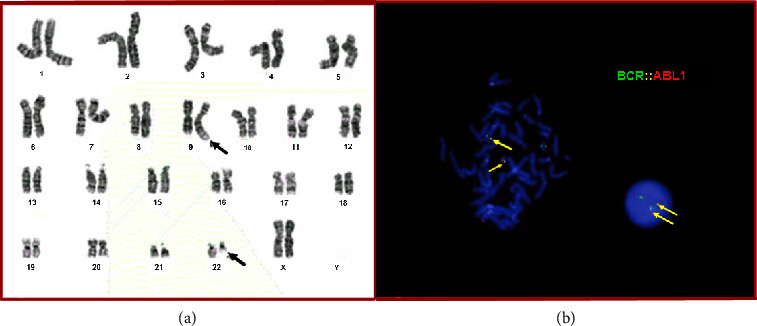
Philadelphia chromosome formation. (a) G-banded metaphases were prepared from unstimulated bone marrow cultures showing the *t*(9;22)(*q*34;*q*11.2) (black arrows). (b) FISH analysis showing BCR::ABL1 fusion (yellow arrows) in both interphase and metaphase cells using a tri-color (aqua not shown) dual fusion translocation probe (Abbott Molecular).

The current specimen leading to the diagnosis of CML had an abnormal karyotype, 46,XX,*t*(9;22)(*q*34;*q*11.2) [20/20] (Figure 1(a)), with the typical reciprocal 9;22 translocation as the sole abnormality in all twenty metaphases analyzed, and the BCR::ABL1 fusion was found in 99.5% of interphases by FISH (Figure 1(b)).

**Figure 2:**
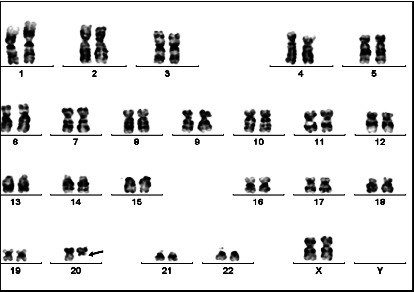
G-banded metaphase chromosome preparations from unstimulated overnight bone marrow cultures from eight years ago. This patient had a 20*q* deletion (arrow) as the sole abnormality in all twenty bone marrow metaphase cells analyzed. Note: *t*(9;22)(*q*34;*q*11.2) was not observed in any of the metaphases at that time.

Bone marrow karyotype analysis eight years prior had revealed a 20*q* deletion as the sole abnormality present in all twenty metaphase cells analyzed (Figure 2). The *t*(9;22)(*q*34;*q*11.2) was not observed in any of the metaphases analyzed at this time.

### 3.2. FISH Studies of Archived Bone Marrow Cytogenetic Pellets

For quality assurance purposes, archived bone marrow cytogenetic pellets were retrieved from the −80°C freezer to confirm the previous karyotype and to look for evidence of the newly discovered BCR::ABL1 fusion since this was not suspected or tested for in the original specimen.

Pellets were thawed and resuspended in fresh Carnoy's fixative for 30 minutes. No modifications from standard protocols were necessary using the archived cytogenetic fixed cell pellets.

**Figure 3:**
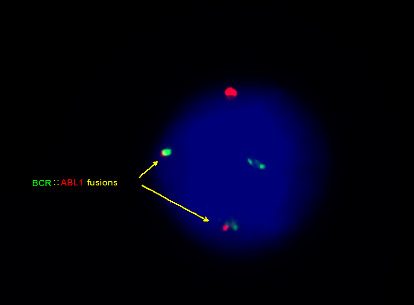
Retrospective cytogenetic analysis. Eight-year-old archived bone marrow pellets were used for BCR::ABL1 FISH studies using a tri-color, dual fusion translocation probe (aqua not shown) (Abbott Molecular). Two yellow (R/G) fusion signals located in the nucleus (arrows) confirming the BCR::ABL1 fusion.

FISH analysis of the archived cytogenetic fixed bone marrow cell pellets from this patient revealed the presence of BCR::ABL1 fusion in 1.8% (9/500) interphase cells (Figure 3).

## 4. Discussion

This patient, who was recently diagnosed with CML, had archived bone marrow cytogenetic pellets available from a previous bone marrow biopsy that were used for retrospective BCR::ABL1 FISH studies. Blood and bone marrow nuclear pellets are routinely prepared and preserved in Carnoy's fixative in clinical cytogenetic labs where they are kept for possible later reference [14, 15]. These pellets can be stored at −80°C where they can be preserved virtually indefinitely.

While fixed cell pellets are not amenable to gene expression studies, such archival material has been shown to be suitable for fluorescence in situ hybridization (FISH) [15, 16]. Satisfactory hybridization signals have been achieved using archived blood and bone marrow samples, but the hybridization efficiency is dependent on the age of the material, with older samples (≥20 years old) performing poorer than fresher ones [17]. More recently, studies have assessed the suitability of archival methanol-fixed bone marrow and blood smears for diagnosing CML by interphase FISH [18]. Thus, archived material is an invaluable resource with potential research and clinical applications. Tapping into this generally unexploited repository of archived cytogenetic material could further retrospective biomarker research in hematological malignancies. This specimen source could augment biobanks that more often store conventional frozen and fixed tissue specimens.

To our knowledge, a retroactive cytogenetic approach using archived Carnoy's fixed nuclear pellets for tracking the origin and growth of leukemia has not been reported in the literature. The success of such an approach, as demonstrated in our case, could be applied to the study of many other cancers.

There are a few reports showing extremely low levels of BCR::ABL1 fusion transcripts resulting from the *t*(9;22) translocation in healthy individuals without apparent oncogenic consequences [19, 20]. It is worth mentioning that, prior to 1995, the Philadelphia chromosome had never been identified in normal subjects. In a study conducted by Biernaux and colleagues, 23 of 117 healthy subjects were positive for BCR-ABL1 mRNA ranging from ∼5 to ∼20 copies in 100 million or more white blood cells [19]. BCR-ABL1 expression was found only in adults, not in umbilical cord blood cells, supporting the idea that normal cells evolve progressively to a neoplastic state over time [21]. Subsequent studies have shown transcripts in umbilical cord samples, but still demonstrated a clear increase with age [22].

It is undeniable that quantitative RT-PCR has greatly improved molecular diagnostic practices. In fact, qRT-PCR and digital PCR are the gold-standard methods to detect BCR-ABL1 transcripts and are extremely sensitive tests for CML diagnosis and monitoring of treatment response. However, these highly sensitive two-step nested reverse transcription PCR (RT-PCR) procedures are prone to high rates of false positive results often caused by contamination of the sample [20, 23], RNA degradation, and other preanalytical factors [24].

Multiple mRNA transcripts detected in gene expression studies might be derived from a single abnormal cell that does not represent a clonal process. FISH represents an opportunity to unambiguously visualize an abnormal clonal population. Use of the highly sensitive and specific tri-color dual fusion FISH probe virtually eliminates detection of false positive cells (i.e., an additional aqua signal is absent in true fusions). No true fusion positive cells were found in normal bone marrow specimens during validation of this probe in our laboratory, and we have established a normal cutoff value less than 1%. Therefore, detection of nine authentic positive cells (1.8%) from our patient's archived specimen represented a true clonal population that was present years before the onset of CML.

Cancer develops through a process of somatic evolution. Mutations in cancer-associated genes drive tumor outgrowth, but our knowledge of the timing of driver mutations and subsequent clonal dynamics is limited. The seed and soil hypothesis proposes that the genetic alteration that leads to the formation of the BCR::ABL1 fusion gene (the “seed”) may occur many years before the onset of symptoms [8, 25]. The “soil” refers to the microenvironment in the bone marrow where the leukemic stem cells reside. The environmental conditions required for the manifestation of CML are not fully understood, but it is thought to involve several factors including mutations and epigenetic changes [25].

It is interesting to note that the del(20*q*) clone detected in the original specimen (before the diagnosis of CML) was completely absent by both karyotype and FISH in the most recent specimen. This suggests that the del(20*q*) was not involved in the neoplastic process leading to CML. It is now accepted that many hematological cancers have a “preleukemic” or prediagnostic stage, such as monoclonal B-cell lymphocytosis in CLL and MGUS in multiple myeloma. It is tempting to suggest that CML may also have an earlier stage not yet recognized with no leukemic presentation.

## 5. Conclusions

This case demonstrates the feasibility of using archived cytogenetic pellets for retrospective cancer studies. It also emphasizes the potential for long latency periods between the occurrence of the BCR::ABL1 fusion and the onset of symptoms. With tyrosine kinase inhibitors being widely available to target the abnormal BCR::ABL1 fusion protein that causes uncontrolled cell growth, future research should include long-term follow-up of asymptomatic patients who harbor the BCR::ABL1 fusion gene to try to identify individuals who are more likely to progress to CML and thus warrant early treatment.

## Data Availability

Data from this case report are unfortunately not available, due to privacy considerations. Data sharing is not applicable to this article, as no datasets were generated or analyzed during the current study.
